# Transmission of Human Enterovirus 85 Recombinants Containing New Unknown Serotype HEV-B Donor Sequences in Xinjiang Uighur Autonomous Region, China

**DOI:** 10.1371/journal.pone.0055480

**Published:** 2013-01-31

**Authors:** Qiang Sun, Yong Zhang, Shuangli Zhu, Huifang Tian, Guohong Huang, Hui Cui, Xiaolei Li, Dongmei Yan, Zhen Zhu, Jing Li, Peng Zheng, Huafang Jiang, Bo Zhang, Xiaojuan Tan, Hui Zhu, Hongqiu An, Wenbo Xu

**Affiliations:** 1 WHO WPRO Regional Polio Reference Laboratory, Ministry of Health Key Laboratory of Medical Virology and Viral Disease, National Institute for Viral Disease Control and Prevention, Chinese Center for Disease Control and Prevention, Beijing, People's Republic of China; 2 Shijiazhuang Center for Disease Control and Prevention, Shijiazhuang, Hebei, People's Republic of China; 3 Xinjiang Uygur Autonomous Region Center for Disease Control and Prevention, Urumqi, Xinjiang Uygur Autonomous Region, People's Republic of China; University of Florida, United States of America

## Abstract

**Background:**

Human enterovirus 85 (HEV85), whose prototype strain (Strain BAN00-10353/BAN/2000) was isolated in Bangladesh in 2000, is a recently identified serotype within the human enterovirus B (HEV-B) species. At present, only one nucleotide sequence of HEV85 (the complete genome sequence of the prototype strain) is available in the GenBank database.

**Principal Findings:**

In this study, we report the genetic characteristics of 33 HEV85 isolates that circulated in the Xinjiang Uighur autonomous region of China in 2011. Sequence analysis revealed that all these Chinese HEV85 isolates belong to 2 transmission chains, and intertypic recombination was found with the new unknown serotype HEV-B donor sequences. Two HEV85 isolates recovered from a patient presenting acute flaccid paralysis and one of his contacts were temperature-insensitive strains, and some nucleotide substitutions in the non-coding regions and in the *2C* or *3D* coding regions may have affected the temperature sensitivity of HEV85 strains.

**Conclusions:**

The Chinese HEV85 recombinant described in this study trapped a new unknown serotype HEV-B donor sequence, indicating that new unknown HEV-B serotypes exist or circulate in Xinjiang of China. Our study also indicated that HEV85 is a prevalent and common enterovirus serotype in Xinjiang.

## Introduction

Human enteroviruses (HEV) are members of the genus *Enterovirus* within the family *Picornaviridae*, order *Picornavirales*, and contains 4 species: HEV-A, HEV-B, HEV-C, and HEV-D [1]. Human enterovirus 85 (HEV85) belongs to the species HEV-B, which currently consists of 60 serotypes, including echovirus (ECHO, serotypes 1–7, 9, 11–21, 24–27, 29–33), coxsackievirus group A (CVA, serotypes 9), coxsackievirus group B (CVB, serotypes 1–6), recently identified HEV serotypes to be designated HEV69, HEV73–75 [2]–[5], HEV77–88 [6]–[9], HEV93 [10], HEV97–98 [9], [11], HEV100–101 [9], HEV106–107 [12], HEV110 [13], and the simian enterovirus SA5. HEV-B viruses cause a variety of diseases such as acute aseptic meningitis, acute flaccid paralysis (AFP), hand, foot, and mouth disease, and acute myocarditis, and others [14]–[17].

The genome of HEV (approximately 7,450 nucleotides) is positive-sense, single-stranded, and contains a long open reading frame (ORF) flanked by a 5′-untranslated region (UTR) and a 3′-UTR. The 5′-UTR is about 740 nucleotides in length and comprises a secondary structure named the internal ribosome entry site [18] which is involved in the replication and internal initiation of translation of the genomic RNA [19]. A single polyprotein translated from the RNA strand is first cleaved into 3 polyprotein precursors: P1, P2, and P3. The P1 protein encodes the 4 structural polypeptides, VP1–VP4, while P2 and P3 are precursors of the nonstructural proteins 2A–2C and 3A–3D, respectively. A short 3′-UTR, approximately 100 nucleotides in length, is located between the ORF and the poly (A) stretch, and comprises structural domains involved in RNA replication [20].

HEV85 is a newly identified serotype within the species HEV-B. Its prototype strain (BAN00-10353/BAN/2000) was identified in a stool sample from a patient presenting with AFP in Bangladesh in 2000, and at present, only one nucleotide sequence of HEV85 (the complete genome sequence of the prototype strain) is available in the GenBank database [9].

In the present study, we analyzed 33 complete *VP1* nucleotide sequences (5 of which also have the complete genome sequences) of the HEV85 isolates recovered from one AFP patient and 32 of his health contacts in the Hotan and Kashgar prefectures, in the southern part of the Xinjiang Uighur autonomous region of China, in 2011. The results indicate that Chinese HEV85 strains are all recombinants with a new unknown serotype HEV-B and circulate in the southern part of Xinjiang.

## Results

### Transmission of HEV85 in Xinjiang in 2011

**Figure 1:**
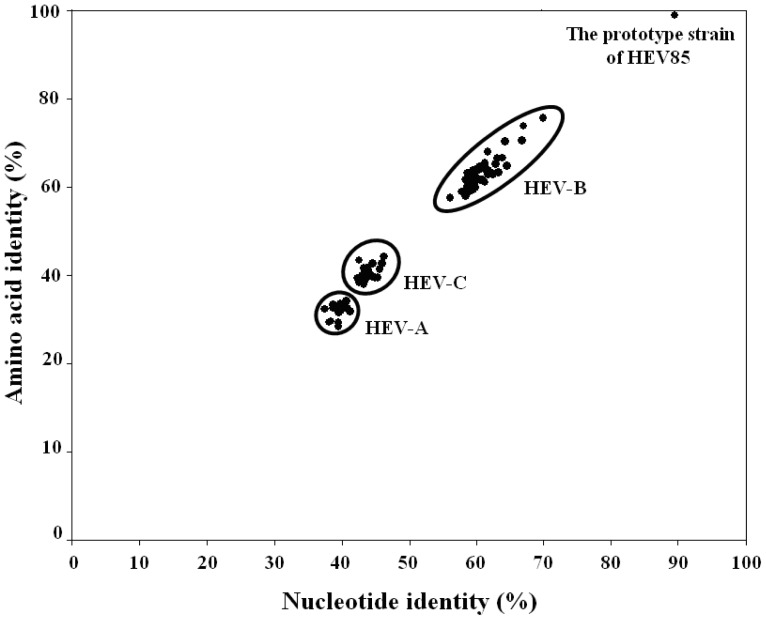
Scatter diagram of the amino acid sequence identity versus nucleotide sequence identity of Chinese HEV85 strain. Analysis of pairwise comparisons between the representative Chinese HEV85 strain, HT-LYKH202F/XJ/CHN/2011, and the prototype strains of other human enterovirus serotypes by comparison of VP1 amino acid sequence identity plotted versus *VP1* nucleotide sequence identity.

The *VP1* region nucleotide sequence alignment included all 33 Chinese HEV85 strains and the prototype strain. The pairwise distance among the 33 Chinese HEV85 strains ranged from 0.000 to 0.026 divergence, and from 0.115 to 0.128 divergence compared with the prototype strain. On the basis of the high nucleotide and amino acid identities of the *VP1* region of all 33 Chinese HEV85 isolates, strain HTYT-ARL-AFP02F, which was isolated from an AFP patient, was selected as the representative strain and depicted the scatter diagram, the plot of the amino acid sequence identity versus nucleotide sequence identity (Figure 1). HEV-D was omitted in this figure because few HEV-D sequences are available in the GenBank database. The plotting curve showed obvious grouping among HEV-A, HEV-B, and HEV-C, and the nucleotide and amino acid sequences of the strain HTYT-ARL-AFP02F had the lowest similarity values with HEV-A and HEV-C species. Intermediate values were observed with HEV-B species, ranging from 55.9% to 69.7% and from 57.3% to 75.2% for nucleotide and amino acid sequences, respectively (Table 1), which confirmed it was a member of HEV-B species. Finally, there were high nucleotide and amino acid sequence similarities values (89.4% and 98.6% for the nucleotide and amino acid sequences, respectively) between strain HTYT-ARL-AFP02F and the HEV85 prototype strain.

**Table 1:** Pairwise nucleotide and amino acid sequence identities between human enterovirus 85 strains and prototype strains of the HEV-B species.

Region	Nucleotide identity (%) [Amino acid identity (%) ]									
	HT-LYKH202F		HTYT-ARL-AFP02F		HTYT-ARLH403F		HTPS-MJH21F		HTPS-MKLH04F	
	Prototype of HEV85	Prototypes of other HEV-B	Prototype of HEV85	Prototypes of other HEV-B	Prototype of HEV85	Prototypes of other HEV-B	Prototype of HEV85	Prototypes of other HEV-B	Prototype of HEV85	Prototypes of other HEV-B
*5′-UTR*	91.5	79.6–88.4	91.3	79.4–88.3	91.2	79.5–88.2	91.3	79.3–88.3	91.2	79.2–88.2
*VP4*	89.3 (100)	69.0–81.1 (75.3–95.6)	89.8 (100)	68.5–81.1 (75.3–95.6)	89.8 (98.5)	68.5–81.1 (75.3–94.2)	89.8 (100)	69.0–81.6(75.3–95.6)	90.3 (100)	69.0–80.6 (75.3–95.6)
*VP2*	89.5 (98.8)	64.9–72.5 (75.5–83.2)	89.2 (98.4)	64.9–72.2 (75.5–83.2)	89.6 (98.8)	64.5–72.2 (75.5–83.2)	89.1 (98.8)	65.0–72.9 (75.5–83.5)	89.6 (98.4)	64.9–72.4 (75.5–83.2)
*VP3*	90.0 (98.7)	63.1–71.0 (66.9–81.9)	89.9 (98.7)	63.1–71.1 (66.9–81.9)	88.9 (99.1)	63.2–70.4 (66.9–81.9)	89.3 (98.7)	62.8–70.7 (66.9–81.5)	89.9 (99.1)	63.1–70.7 (66.9–81.9)
*VP1*	89.4 (98.9)	55.5–69.8 (57.3–75.6)	89.4 (98.6)	55.9–69.7 (57.3–75.2)	89.1 (98.6)	55.6–69.0 (57.0–75.6)	89.0 (97.9)	55.5–69.7 (57.0–75.2)	89.6 (98.9)	55.6–69.1 (57.3–75.6)
*2A*	79.3 (94.6)	75.1–81.7 (84.6–96.0)	79.5 (94.6)	74.8–81.5 (84.6–96.0)	79.7 (94.6)	75.3–81.1 (84.6–96.0)	80.2 (95.3)	74.8–81.5 (85.3–96.6)	80.0 (94.6)	75.5–81.7 (84.6–96.0)
*2B*	80.1 (94.9)	75.7–83.8 (93.9–98.9)	80.4 (94.9)	75.0–83.5 (93.9–98.9)	80.1 (94.9)	75.0–83.5 (93.9–98.9)	80.8 (94.9)	76.0–83.1 (93.9–98.9)	79.4 (94.9)	75.0–83.1 (93.9–98.9)
*2C*	83.8 (98.4)	79.2–84.9 (96.3–99.0)	84.3 (98.7)	79.4–84.9 (96.6–99.3)	84.1 (99.0)	79.5–85.2 (96.9–99.6)	84.3 (98.7)	79.6–85.1 (96.6–99.3)	84.0 (97.8)	79.1–84.7 (85.7–99.0)
*3A*	80.1 (97.7)	75.6–85.3 (91.0–98.8)	80.5 (97.7)	76.0–85.7 (91.0–98.8)	80.5 (97.7)	76.0–85.7 (91.0–98.8)	79.4 (97.7)	74.9–84.6 (91.0–98.8)	80.1 (97.7)	75.6–85.3 (91.0–98.8)
*3B*	84.8 (100)	69.6–87.8 (90.9–100)	87.8 (100)	72.7–87.8 (90.9–100)	87.8 (100)	72.7–87.8 (90.9–100)	86.3 (100)	71.2–89.3 (90.9–100)	86.3 (100)	71.2–89.3 (90.9–100)
*3C*	83.9 (98.9)	75.9–85.7 (92.8–99.4)	83.7 (98.3)	75.9–85.6 (92.3–98.9)	83.9 (98.9)	76.1–85.7 (92.8–99.4)	84.3 (98.9)	76.1–86.1 (92.8–99.4)	84.1 (98.9)	75.7–85.4 (92.8–99.4)
*3D*	84.9 (97.6)	77.5–85.6 (95.0–98.2)	85.5 (98.0)	78.1–86.1 (95.4–98.8)	85.4 (97.8)	77.8–86.2 (95.2–98.4)	85.1 (97.8)	77.7–85.7 (95.2–98.4)	85.6 (98.0)	77.9–86.0 (95.4–98.7)
*3′-UTR*	87.5	74.0–91.3	87.5	75.0–91.3	88.4	74.0–92.3	87.5	73.0–91.3	88.4	74.0–92.3

**Figure 2:**
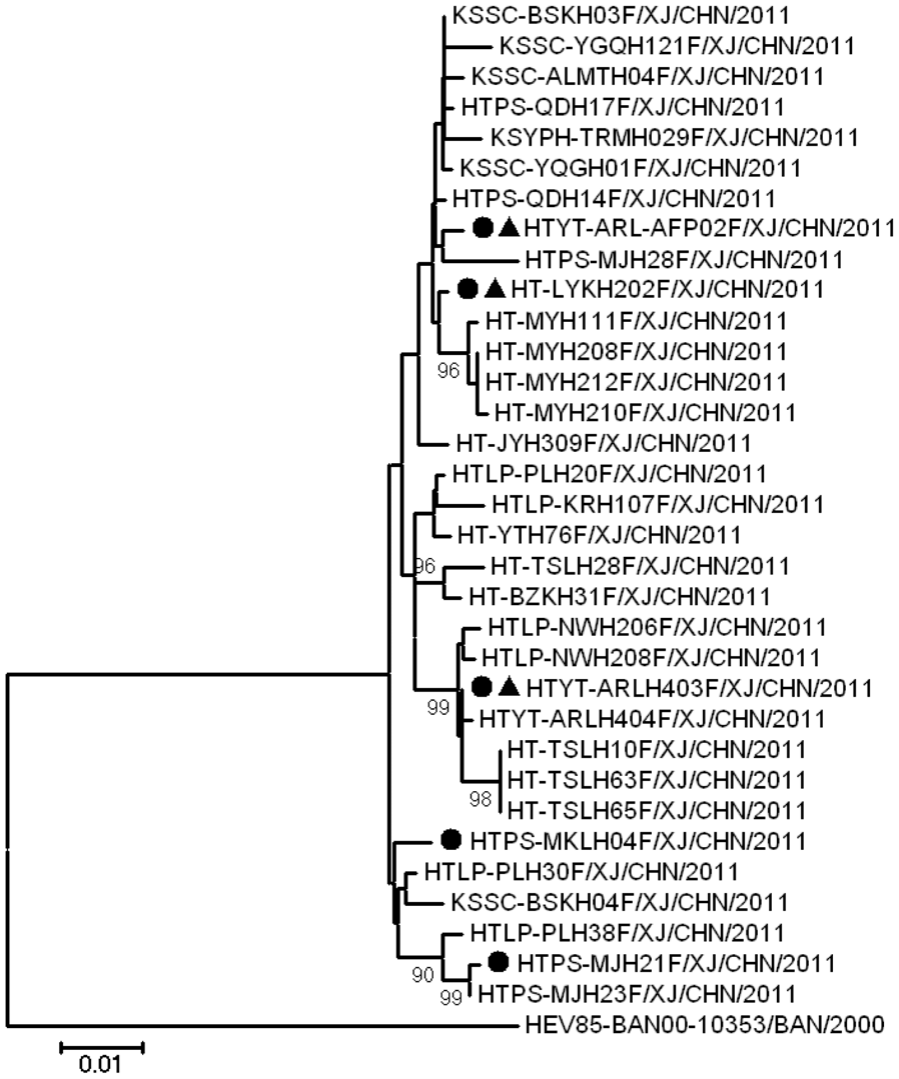
Transmission of the Chinese HEV85 strains in Xinjiang of China. Phylogenetic analysis and genetic characterization of 33 Chinese HEV85 isolates. Strains indicated by the symbol • indicate HEV85 strains that were subjected to complete genome sequencing; strains indicated by the symbol ▴ indicate HEV85 strain that were subjected to the temperature sensitivity test.

Phylogenetic analysis was performed on the basis of the alignment of *VP1* region sequences described above (Figure 2). Clearly all 33 Chinese HEV85 strains are derived from the same origin, and a 2.0% nucleotide divergence was found among these strains. They were all circulated in the Hotan and Kashgar prefectures of southern Xinjiang from September to November 2011,

### Full-length genomic characterizations of Chinese HEV85 strains

The full-length genome sequences of 5 selected Chinese HEV85 strains were determined (randomly selected on the basis of their genetic relationships; Figure 2). The results showed that they were similar to the only reported genome of HEV85 (the prototype strain), with 7,422–7,424 nucleotides, including a 5′-UTR of 742–744 nucleotides (0–2 nucleotide insertion compared with the prototype strain), a single ORF of 6,579 nucleotides encoding a single polyprotein of 2,191 amino acids, and a 3′-UTR of 101 nucleotides preceding the poly (A) tract. All these sequences shared 98.34–99.08% nucleotide sequence identities with each other, validating the circulation of HEV85 in Xinjiang of China.

A comprehensive comparison of nucleotide sequence and deduced amino acid sequence identities between the 5 Chinese HEV85 strains and the prototype strain of HEV-B viruses including HEV85 is shown in Table 1. The nucleotide sequence identities between the 5 Chinese HEV85 strains and the prototype HEV85 strain were 86.46–86.73% in the full-length genome sequence and 89.28–89.67%, 82.06–82.70%, and 84.15–84.68% in the *P1*, *P2*, and *P3* coding regions, respectively. The deduced amino acid sequence identities between the 5 Chinese HEV85 strains and the prototype HEV85 strain were 98.60–98.95%, 96.54%–97.23%, and 98.02–98.28% in the *P1*, *P2*, and *P3* coding regions, respectively. Interestingly, in the capsid region, the nucleotide sequences of the 5 Chinese HEV85 isolates were more closer to the HEV85 prototype strain (89.28–89.67%) than to other HEV-B prototype strains (58.8–68.3%, Table 1); while in the noncapsid region, the nucleotide sequences of the 5 Chinese HEV85 isolates and all HEV-B viruses including HEV85 were almost equidistant from each other (74.3–81.8%) and did not cluster with regard to all known serotypes HEV-B viruses, which indicated the occurrence of recombination events in the noncapsid region.

### Chinese HEV85 strains recombined with new unknown serotype HEV-B donor sequences

**Figure 3:**
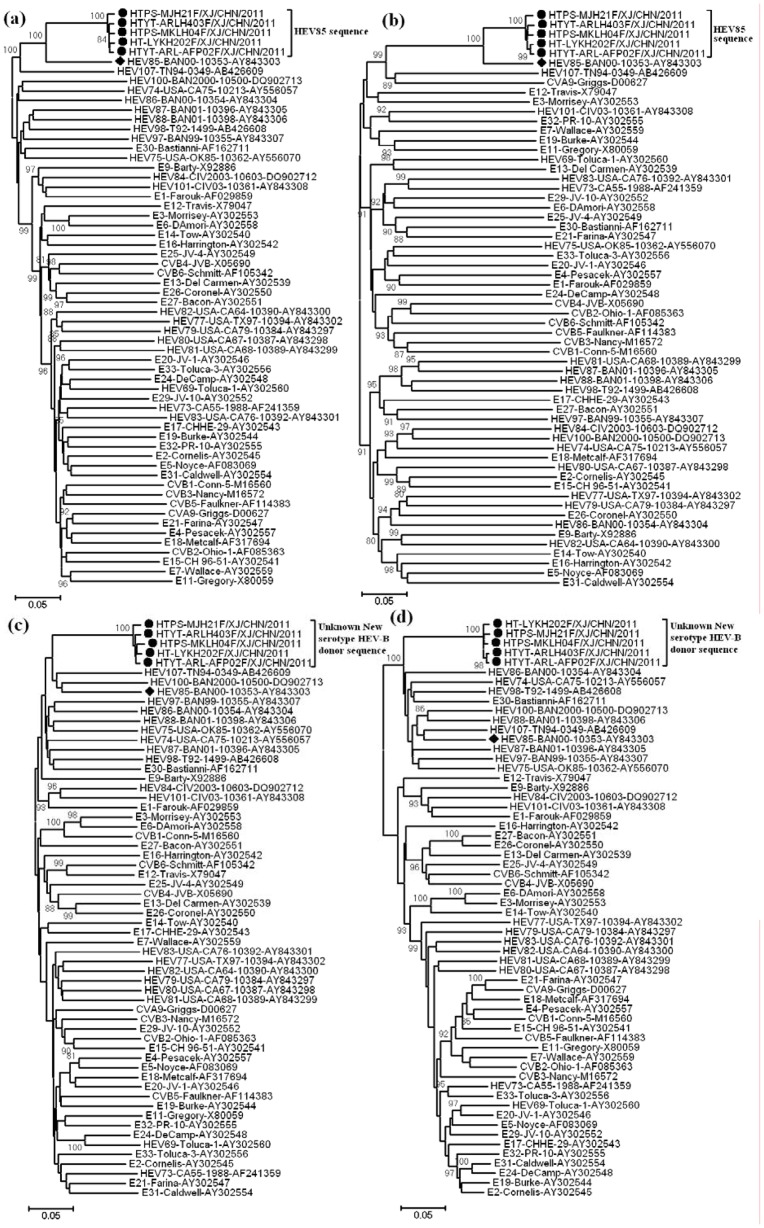
Unrooted trees representing the phylogenetic relationships among Chinese HEV85 strains and other members of HEV-B. The phylogenetic trees based on nucleotide sequences for *VP1* coding sequences (a), *P1* coding sequences (b), *P2* coding sequences (c), and *P3* coding sequences (d) were constructed from the nucleotide sequence alignment using the neighbor-joining algorithm of the MEGA 5.0 software. Numbers at nodes indicate bootstrap support for that node (percent of 1000 bootstrap pseudoreplicates). The scale bars represent the genetic distance, and all unrooted trees have the same scale.

Alignments of the *VP1*, *P1*, *P2*, *and P3* region nucleotide sequences were carried out among the 5 selected Chinese HEV85 strains described above and the prototype strains of HEV-B, and phylogenetic trees were constructed (Figure 3). The phylogenetic tree analysis suggested that Chinese HEV85 strains are monophyletic in the *VP1* region, and all 5 Chinese HEV85 strains clustered together with the HEV85 prototype stain (Figure 3a), confirming the classification of these isolates as a single enterovirus type. All 5 Chinese HEV85 strains clustered with the HEV85 prototype strain in the *P1* capsid region (Figure 3b), but not in the *P2* and *P3* noncapsid regions (Figure 3c and 3d), indicating that recombination occurred among Chinese HEV85 strains.

**Figure 4:**
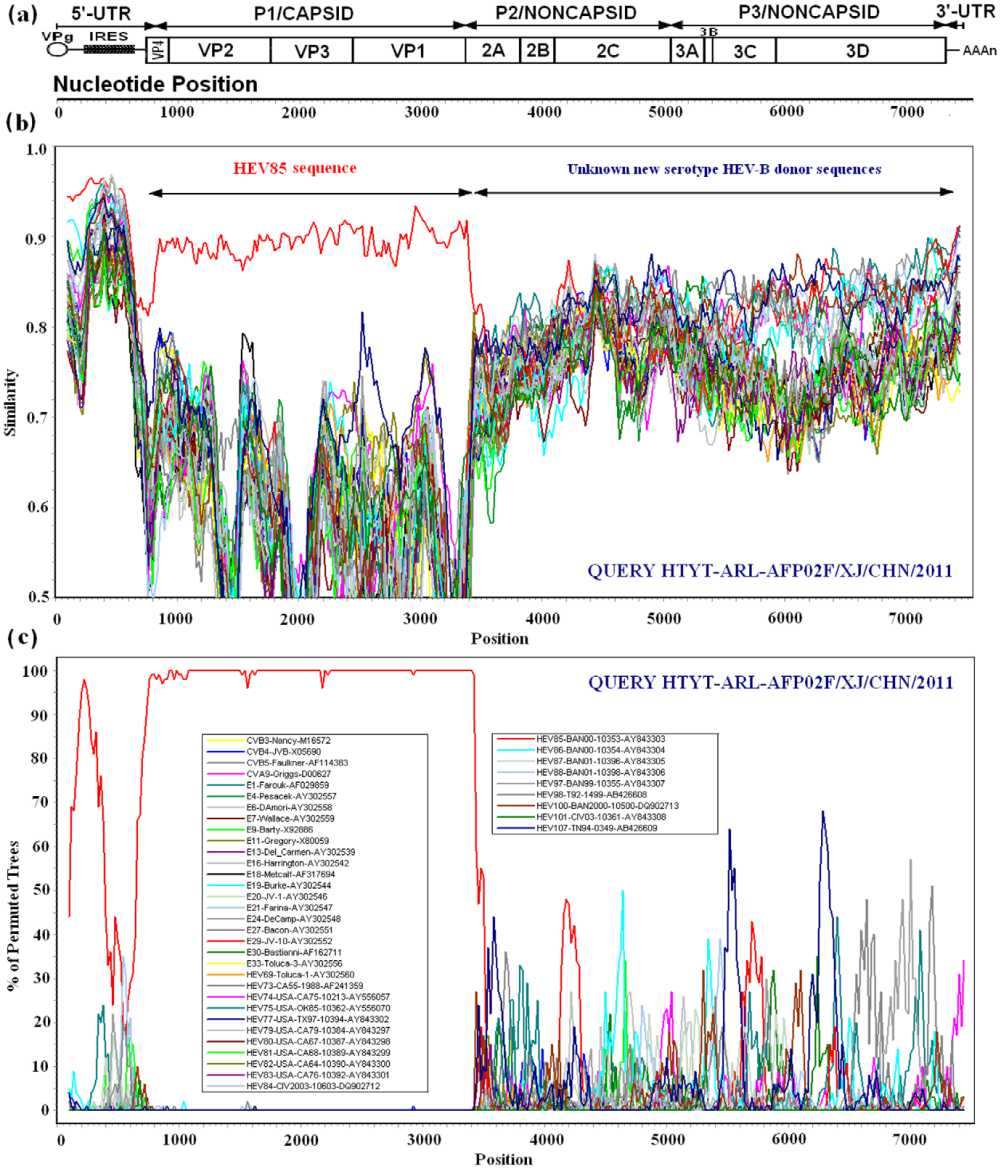
Similarity plot and bootscan analysis of whole genome of Chinese HEV85 strains. Gene structure organization (a), similarity plot (b), and bootscan analysis (c) of complete HEV-B genomes using a sliding window of 200 nt moving in 20 nt steps. The HTYT-ARL-AFP02F/XJ/CHN/2011 isolate was used as a query sequence and is indicated in the lower right corner, and for each bootscan analysis, the names of viruses of the query sequence are indicated in the upper right corner.

Similarity plot and bootscan analyses revealed recombination between the Chinese HEV85 strains and HEV-B strains at the *2A*–*2B* junction. The Chinese HEV85 strains were all identified as a HEV85 capsid sequence containing an unidentified sequence in the *P2* and *P3* coding regions that was apparently not related to those of the HEV85 strains (Figure 4). Nucleotide and amino acid sequences in the *P2* and *P3* regions are highly conserved within an enterovirus species [21], and *P2* and *P3* sequences do not correlate with HEV serotypes due to frequent recombination; however, these sequences clearly distinguish different HEV species [9]. Comparison of the *P2* and *P3* coding region sequences of the Chinese HEV85 strains with those of certain prototype strains of HEV-A, B, C, and D revealed no sequence match above 84.68%, and showed higher similarity to HEV-B than to HEV-A, C, and D. In addition, the deduced amino acid sequence of the recombinant noncapsid sequences of the Chinese HEV85 strains showed high identity with HEV-B, especially those of prototype HEV88 (97.9%), prototype HEV75 (97.8%), and prototype HEV85 (97.6%). These results confirmed that the recombinant noncapsid sequences were classified into the HEV-B phylogeny (Figure 4).

Because the nucleotide sequence identities between the Chinese HEV85 strain and all the HEV-B prototypes in the noncapsid region ranged from 78.63% (HEV80) to 84.30% (HEV107), the noncapsid donor sequences could not be classified as any known serotype; the donor sequences were from a new unknown serotype within HEV-B.

### Temperature sensitivity

**Figure 5:**
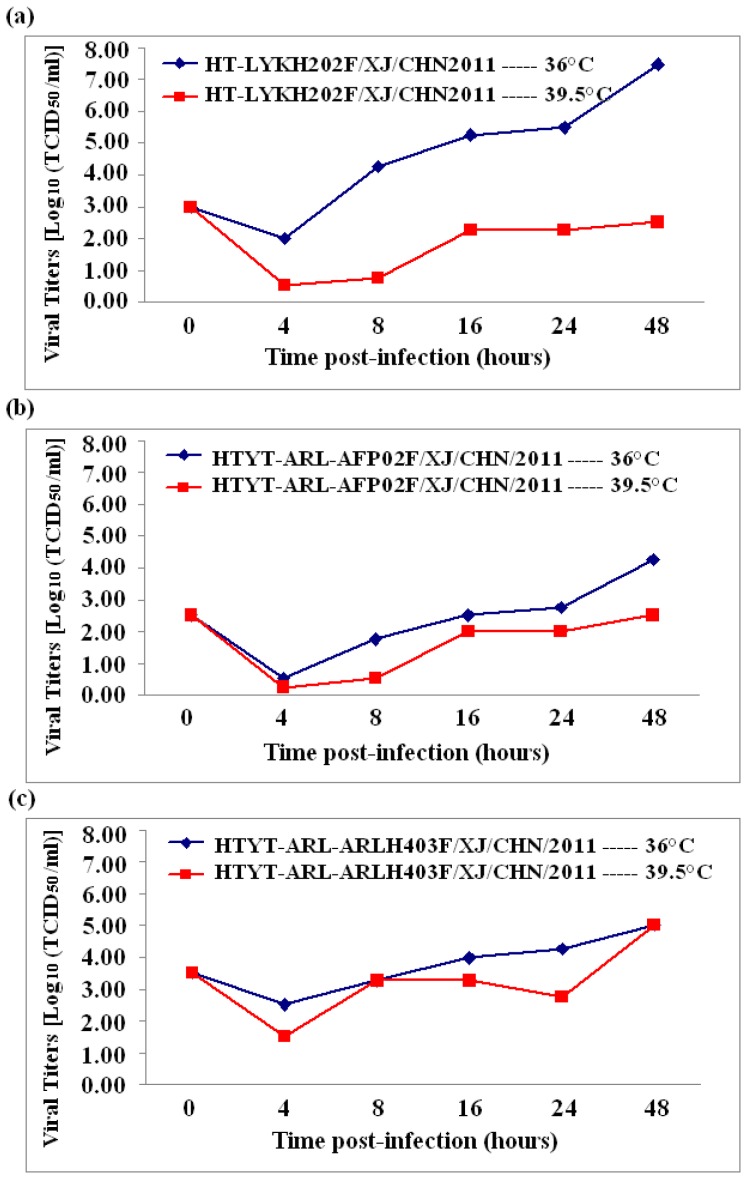
Temperature sensitivity test curves of 3 selected HEV85 isolates. The blue and red lines represent the growth trend of the viruses on RD cell at 36°C and 39.5°C, respectively.

Three selected Chinese HEV85 isolates (HT-LYKH202F, HTYT-ARL-AFP01F, and HTYT-ARLH403F) were compared to each other with regard to replication capacity at an elevated temperature (39.5°C), and showed different temperature sensitivities (Figure 5). Strain HT-LYKH202F was temperature-sensitive with titer reduction of more than 2 logarithms at 36°C/39.5°C, whereas the other 2 strains showed definitely lower temperature sensitivities (titers reduced less than 2 logarithms at 36°C/39.5°C). This indicates that the replication efficiencies of these strains remain the same even at elevated temperatures (Figure 5).

In order to further investigate determinants of temperature sensitivity among these strains, differences in nucleotide and amino acid sequences between the temperature-sensitive strain and the 2 temperature-insensitive strains were summarized (Table 2). The results indicate that the numbers of different nucleotides and amino acid were 46 and 6, respectively. On the basis of the above results and on many previous studies of other serotypes of HEVs [22], [23], we believe that a total of 8 nucleotides in the *5′-UTR* (nt161), *2C* (nt4102 and nt4256), *3D* (nt6178, nt6340, nt6719, and nt6972), and *3′-UTR* (nt7330) regions are candidate determinant sites for temperature sensitivity in HEV85.

**Table 2:** Sites of nucleotide and amino acid disparity between the temperature-sensitive strain HT-LYKH202F (a) and temperature-insensitive strains HTYT-ARL-AFP01F (b) and HTYT-ARLH403F (c).

Origin or donor	Region	Nucleotide				Amino acid			
		Position	Strain (a)	Strain (b)	Strain (c)	Position	Strain (a)	Strain (b)	Strain (c)
**HEV85 Sequence**	***5*** *′-* ***UTR***	**161**	**C**	**T**	**T**	**/**	**/**	**/**	**/**
	***VP4***	**/**	**/**	**/**	**/**	**/**	**/**	**/**	**/**
	***VP2***	989	C	T	T				
	***VP3***	1985	C	T	T				
		2174	T	C	C				
	***VP1***	2729	A	T	T				
		2915	C	T	T				
**New unknown serotype HEV-B donor sequence**	***2A***	3449	C	T	T				
		3551	A	G	G				
		3623	T	C	C				
	***2B***	3929	C	T	T				
		3953	T	C	C				
	***2C***	**4102**	**G**	**A**	**A**	**1120**	**Ser**	**Asn**	**Asn**
		**4256**	**T**	**A**	**A**	**1171**	**Asp**	**Glu**	**Glu**
		4565	T	C	C				
		4721	C	T	T				
		4799	T	C	C				
		4898	C	T	T				
		4910	T	C	C				
		4976	T	C	C				
		5006	T	C	C				
	***3A***	5081	T	C	C				
	***3B***	5354	G	A	A				
		5378	G	A	A				
	***3C***	5549	T	C	C				
		5576	C	T	T				
		5630	A	G	G				
		5645	G	A	A				
		5696	T	C	C				
		5882	A	T	T				
	***3D***	5999	G	A	A				
		6113	C	T	T				
		**6178**	**C**	**T**	**T**	**1812**	**Ala**	**Val**	**Val**
		6212	A	G	G				
		6252	T	C	C				
		6267	T	C	C				
		6284	T	G	G				
		**6340**	**G**	**A**	**A**	**1866**	**Arg**	**Lys**	**Lys**
		6350	T	A	A				
		6431	G	A	A				
		**6719**	**C**	**A**	**A**	**1992**	**Asp**	**Glu**	**Glu**
		6725	T	C	C				
		6761	T	C	C				
		**6972**	**A**	**G**	**G**	**2077**	**Thr**	**Ala**	**Ala**
		7175	C	T	T				
		7244	T	C	C				
	***3*** *′-* ***UTR***	**7330**	**T**	**C**	**C**	**/**	**/**	**/**	**/**

Nucleotide and amino acid positions in shadow indicate candidate determinant sites for temperature sensitivity in HEV85.

## Discussion

Molecular serotyping methods have enabled the rapid identification of new HEV serotypes that are untypeable by traditional neutralization of virus isolates in cell culture using standardized antisera, and as a result, sequencing of the complete *VP1* capsid region has emerged as the gold standard for HEV typing to distinguish serotypes [24]–[26]. Molecular identification of recently identified HEV serotypes, including HEV85, provides a tool to assist in the epidemiological investigation of AFP cases that are associated with non-polio enterovirus infection.

In this study, we identified 33 HEV85 isolates in the Hotan and Kashgar prefectures in the Xinjiang region in China from 6^th^ Aug to 20^th^ Sep 2011, which indicated the prevalence of this serotype of HEV in this region. These 2 prefectures are located in the southernmost part of Xinjiang and have great biological and climatic differences due to the large area (409,800 square kilometers in total), which includes the southern mountain area (temperate or cold temperate climate), the oasis plain area (warm temperate), and the northern desert area (continental desert climate). The viruses were isolated from the oasis plain area, where is very suitable for the growth and survival of HEV. HEVs are known to be propagated mainly by fecal-oral transmission, suggesting that the river, the source of water, geographic circumstances, and air humidity are major factors affecting transmission. This is confirmed by the facts that the majority of the prototype strains of novel HEV serotypes were isolated from tropical and subtropical countries and regions such as Bangladesh [9], [27], California (USA) [2], [9], Egypt [28] and Cote d'Ivoire [9], etc, while the prevalence of these viruses seems to be relatively restricted in the temperate countries.

One of the 33 Chinese HEV85 isolates, strain HTYT-ARL-AFP02F, was derived from an adult patient who presented with AFP in July 2011. Although there is no strong evidence to prove that HEV85 is the causative pathogen of AFP, as the virus was recovered from a stool specimen, it showed high transmissibility based on the fact that it has high nucleotide and amino acid identities with other Chinese HEV85 isolates picked from the contacts of this AFP patient. This strain was also temperature-insensitive and could grow at relatively high temperatures, which is usually an indicator of high virulence or transmissibility [23], [29]. These findings suggest that HEV85 is an important human pathogen; however, more data are necessary before HEV85 can be positively associated with any particular human disease.

Strain HT-LYKH202F was a temperature-sensitive strain, whereas strains HTYT-ARL-AFP02F (isolated from an AFP patient) and HTYT-ARLH403F were not. All these 3 strains had high nucleotide and amino acid similarities with each other (a total of 8 nucleotide substitutions of which 6 caused amino acid substitutions), which implies that possibly one or several sites may play significant roles in this biological feature. Our research team is currently using reverse genetics methods to elucidate the mechanism of temperature sensitivity of HEV85, in order to identify several vaccine candidate strains to meet emergency needs in case of epidemic outbreaks triggered by these serotypes in the future.

Recently, increasing numbers of publications have demonstrated that recombination is extremely frequent among HEVs [22], [30], [31]. Therefore, it can be concluded that HEVs exist as a huge reservoir of genetic material comprising a limited quantity of capsid gene sets defining a finite number of serotypes and a range of non-structural genes that recombine frequently to produce new virus variants. The Chinese HEV85 strains identified in this study are no exception, and have recombined with other HEV-B viruses; this is supported by the genomic features, the phylogenetic tree based on the alignments of the *P1*, *P2*, *P3* regions, and the similarity and bootscan plots. Together with the isolation of HEV85, a recently identified novel HEV serotype, the trapping of an unknown new serotype HEV-B donor sequence in the Chinese HEV85 recombinant described in this study suggests that new HEV-B serotypes are in circulation in the Xinjiang region of China. Hence, we have increased HEV surveillance in the Xinjiang region in order to determine the precise donor sequence of the new serotype HEVs.

Identifying HEVs recovered from patient specimens has several implications in the medical field, and HEVs should be characterized to allow epidemiological surveillance of the potential epidemic risks associated with new viruses. Indeed, there are more and more novel HEV serotypes that exhibit some features pertaining to epidemic risk; for example, they were isolated from patients, show high prevalence in many regions, and even elicit epidemic outbreaks [32]–[35]. Therefore, it is considered important to conduct stool surveys in the Xinjiang Autonomous Region, as a few AFP patients have been identified during AFP surveillance activities in support of global polio eradication. However, an effective HEV pathogen surveillance system has yet to be established in mainland China; therefore, current information on HEV85 or other novel HEV serotypes in circulation is not available. Once this is in place, it is expected that monitoring trends in the transmission of HEV85 or other new HEV types will be made possible by molecular epidemiological studies over a wide area. As more laboratories adopt molecular typing methods in order to identify newly described HEV serotypes, it will become possible to address the global distribution of HEV85 and to better understand its epidemiology, disease burden, and spectrum of illness.

## Materials and Methods

### Viruses

This study did not involve human participants or human experimentation; the only human materials used were stool samples collected from AFP patients or their close contacts at the instigation of the Ministry of Health P. R. of China for public health purposes, and written informed consent for the use of their clinical samples was obtained from all patients involved in this study. This study was approved by the second session of the Ethics Review Committee of the Chinese Center for Disease Control and Prevention. The HEV85 strains used in this study were isolated from stool specimens from one AFP patient and 32 of his healthy close contacts in the Hotan and Kashgar prefectures in the Xinjiang region of China. Viruses were isolated from original stool specimens by propagation in human rhabdomyosarcoma (RD) and human larynx carcinoma (HEp-2) cells by conventional methods and then sequenced [36].

### Determination of the complete *VP1* nucleotide sequence

Viral RNA was extracted from the viral isolates using a QIAamp Viral RNA Mini Kit (Qiagen, Valencia, CA, USA). The complete *VP1*/capsid region of each HEV85 strain was amplified by reverse transcription-polymerase chain reaction (RT-PCR) using the primers described previously [9]. RT-PCR was performed with an Access RT-PCR Kit (Promega, Madison, Wisconsin, USA) according to the manufacturer's instructions. The PCR products obtained were purified using the QIAquick Gel extraction kit (Qiagen), and the amplicons were bi-directionally sequenced using an ABI PRISM 3100 Genetic Analyzer (Applied Biosystems, Hitachi, Japan) [37].

### Full-length genome sequencing of Chinese HEV85 strains

First of all, viral RNA was converted to cDNA by a random priming strategy. Then the complete genome sequences of the viruses were acquired according to the published strategies for HEV sequencing [30], [38]. Briefly, the overlapping fragments representing the complete genomes were amplified by RT-PCR with the specific, non-degenerate primers listed in Table 3, and the primer-walking strategy was used to close gaps as necessary. The PCR products obtained were purified for sequencing using the QIAquick Gel extraction kit (Qiagen), and the amplicons were then bi-directionally sequenced using fluorescent dideoxy-chain terminators and an ABI PRISM 3100 genetic analyzer (Applied Biosystems, Foster City, CA, USA). 5′-segment sequences were determined by using a 5′-rapid amplification of cDNA ends core set (Takara Biomedicals, Dalian, China), according to the manufacturer's instructions [30].

**Table 3:** PCR and sequencing primers.

Primer	Nucleotide position (nt)	Primer sequence (5′–3′)	Orientation	Reference
0001S48		GGGGACAAGTTTGTACAAAAAAGCAGGCTTTAAAACAGCTCTGGGGTT	Forward	[43]
HEV85-1049A	1030–1049	ACAACAACATTGGCACACTC	Reverse	This study
HEV85-636S	636–655	GCCATCCGGTGTCAAATAGA	Forward	This study
HEV85-1656A	1637–1656	AGCCTGATCCGTGTAGTCCA	Reverse	This study
HEV85-1487S	1487–1506	TGGGCAACCTAACGATCTTC	Forward	This study
HEV85-2640A	2611–2640	GACTCTGAGCGGGAATGGTA	Reverse	This study
HEV85-2480S	2461–2480	GAAGGGAGAGCGAGCCTAGT	Forward	This study
HEV85-3406A	3406–3425	TAGTCCTCCCAGACGCAACT	Reverse	This study
HEV85-3290S	3268–3287	CGTGCCACCATTAACACAGT	Forward	This study
HEV85-4257A	4238–4257	TTCAATGGTGGCAATCTGAC	Reverse	This study
HEV85-4072S	4072–4091	GGCTGGCTCAAGAAGTTCAC	Forward	This study
HEV85-5062A	5043–5062	CTGGTGGACCTTGGAAAAGA	Reverse	This study
HEV85-4930S	4930–4949	GCCATCCAGTTCATTGACAG	Forward	This study
HEV85-5880A	5861–5880	GTTGCCACCAACGTGTATTC	Reverse	This study
HEV85-5722S	5722–5741	TACATCCCCGTTGGTCAAGT	Forward	This study
HEV85-6619A	6600–6619	GATGGCCGTCAAGCATAACT	Reverse	This study
HEV85-6436S	6436–6455	GTGGCTAAAGGCAAGTCCAG	Forward	This study
HEV85-7260A	7241–7260	TACTGGGACGCTCCTGATCT	Reverse	This study
HEV85-7084S	7084–7103	CAATACCCCTTCCTCGTTCA	Forward	This study
7500A		GGGGACCACTTTGTACAAGAAAGCTGGG(T)_24_	Reverse	[43]

### Phylogenetic and Bioinformatics analyses

The nucleotide and deduced amino acid sequences of the HEV85 isolates were compared to one another and to those of other HEV-B serotypes by pairwise alignment using the MEGA program (version 5.0; Sudhir Kumar, Arizona State University, Tempe, Arizona, USA) [39], [40], and the identity matrices were analyzed by plotting VP1 amino acid identities versus *VP1* nucleotide identities for each virus pair using the SigmaPlot program (version 10.0; Systat Software Inc., San Jose, CA, USA). Phylogenetic trees were constructed by the neighbor-joining method implemented in the MEGA program using the Kimura-2-parameters model. Regions containing alignment gaps were omitted from the analysis. The branch lengths of the dendrogram were determined from the topologies of the trees and were obtained by majority rule consensus among 1000 bootstrap replicates. Bootstrap values greater than 80% were considered statistically significant for grouping.

### Recombination analysis

The nucleotide alignment containing the genome sequence of a Chinese HEV85 strain (HTYT-ARL-AFP02F) and HEV-B prototype strains (ECHO-1, 4, 6, 7, 9, 11, 13, 16, 18–21, 24, 27, 29, 30, 33, CVA9, CVB3–5, HEV69, HEV73–75, HEV77, 79–88, HEV97–98, HEV100–101, and HEV107) was generated using the MEGA program (version 5.0; Sudhir Kumar, Arizona State University, Tempe, Arizona, USA) [39], [40]. Once aligned, similarity plot and bootscan analyses were performed using Simplot program (version 3.5.1; Stuart Ray, Johns Hopkins University, Baltimore, Maryland, USA) [41].

### Assay for temperature sensitivity

Temperature sensitivities of 3 selected HEV85 isolates (HT-LYKH202F, HTYT-ARLH403F and HTYT-ARL-AFP02F) were assayed on monolayer RD cells in 24-well plates as described before [42]. Briefly, the 24-well plates were inoculated with 50 µl of undiluted virus stocks. Two different incubators were used; the temperature of one incubator was adjusted to 36°C (optimal temperature for virus propagation), while the temperature of the other incubator was adjusted at 39.5°C (supraoptimal temperature for virus propagation). After absorption at 36°C or at 39.5°C for 1 h, the unabsorbed virus inoculum was removed, 100 µl of maintenance medium was added to each well, and the plates were continually incubated at 36°C or at 39.5°C, separately. After 5 time points post-infection (4, 8, 16, 24, and 48 h), the plates were harvested, and the cell culture infectious dose 50% (CCID_50_) was calculated by the end-point dilution method on monolayer RD cells in 96-well plates at 36°C. In this study, virus isolates showing more than a 2-logarithm reduction in titer at different temperatures were considered to be temperature-sensitive [22].

### Nucleotide sequence accession numbers

The 5 full-length genomic sequences of HEV85 strains that were determined in this study have been deposited in the GenBank database under the accession numbers JX898905 to JX898909. The 28 other complete *VP1* nucleotide sequences (870 nucleotides) of HEV85 strains that were determined in this study have been deposited in the GenBank database under the accession numbers JX898910 to JX898937.

## References

[1] Knowles NJ, Hovi T, Hyypiä T, King AMQ, Lindberg M, et al.. (2011) Picornaviridae. In: King AMQ, Adams MJ, Carstens EB, Lefkowitz EJ, editors. Diego Virus taxonomy: classification and nomenclature of viruses: Ninth Report of the International Committee on Taxonomy of Viruses. Elsevier. pp. 855–880.

[2] Oberste M, Schnurr D, Maher K, al-Busaidy S, Pallansch M (2001) Molecular identification of new picornaviruses and characterization of a proposed enterovirus 73 serotype. J Gen Virol 82: 409–416. 11161280 10.1099/0022-1317-82-2-409

[3] Norder H, Bjerregaard L, Magnius LO (2002) Open reading frame sequence of an Asian enterovirus 73 strain reveals that the prototype from California is recombinant. J Gen Virol 83: 1721–1728. 12075091 10.1099/0022-1317-83-7-1721

[4] Oberste MS, Michele SM, Maher K, Schnurr D, Cisterna D, et al (2004) Molecular identification and characterization of two proposed new enterovirus serotypes, EV74 and EV75. J Gen Virol 85: 3205–3212. 15483233 10.1099/vir.0.80148-0

[5] Wang J, Zhang Y, Hong M, Li X, Zhu S, et al (2012) Isolation and characterization of a Chinese strain of human enterovirus 74 from a healthy child in the Tibet Autonomous Region of China. Arch Virol 157: 1593–1598. 22576315 10.1007/s00705-012-1332-9

[6] Bailly JL, Cardoso MC, Labbe A, Peigue-Lafeuille H (2004) Isolation and identification of an enterovirus 77 recovered from a refugee child from Kosovo, and characterization of the complete virus genome. Virus Res 99: 147–155. 14749180 10.1016/j.virusres.2003.11.006

[7] Norder H, Bjerregaard L, Magnius L, Lina B, Aymard M, et al (2003) Sequencing of ‘untypable’ enteroviruses reveals two new types, EV-77 and EV-78, within human enterovirus type B and substitutions in the BC loop of the VP1 protein for known types. J Gen Virol 84: 827–836. 12655083 10.1099/vir.0.18647-0

[8] Oberste MS, Maher K, Patterson MA, Pallansch MA (2007) The complete genome sequence for an American isolate of enterovirus 77. Arch Virol 152: 1587–1591. 17497234 10.1007/s00705-007-0978-1

[9] Oberste MS, Maher K, Nix WA, Michele SM, Uddin M, et al (2007) Molecular identification of 13 new enterovirus types, EV79–88, EV97, and EV100–101, members of the species Human Enterovirus B. Virus Res 128: 34–42. 17485128 10.1016/j.virusres.2007.04.001

[10] Junttila N, Leveque N, Kabue JP, Cartet G, Mushiya F, et al (2007) New enteroviruses, EV-93 and EV-94, associated with acute flaccid paralysis in the Democratic Republic of the Congo. J Med Virol 79: 393–400. 17311342 10.1002/jmv.20825

[11] Smura T, Blomqvist S, Paananen A, Vuorinen T, Sobotova Z, et al (2007) Enterovirus surveillance reveals proposed new serotypes and provides new insight into enterovirus 5′-untranslated region evolution. J Gen Virol 88: 2520–2526. 17698662 10.1099/vir.0.82866-0

[12] Yamashita T, Ito M, Tsuzuki H, Sakae K, Minagawa H (2010) Molecular identification of enteroviruses including two new types (EV-98 and EV-107) isolated from Japanese travellers from Asian countries. J Gen Virol 91: 1063–1066. 19955564 10.1099/vir.0.016014-0

[13] Harvala H, Sharp CP, Ngole EM, Delaporte E, Peeters M, et al (2011) Detection and genetic characterization of enteroviruses circulating among wild populations of chimpanzees in Cameroon: relationship with human and simian enteroviruses. J Virol 85: 4480–4486. 21345956 10.1128/JVI.02285-10 PMC3126250

[14] Zhu Z, Xu WB, Xu AQ, Wang HY, Zhang Y, et al (2007) Molecular epidemiological analysis of echovirus 19 isolated from an outbreak associated with hand, foot, and mouth disease (HFMD) in Shandong Province of China. Biomed Environ Sci 20: 321–328. 17948768

[15] dos Santos GP, da Costa EV, Tavares FN, da Costa LJ, da Silva EE (2011) Genetic diversity of Echovirus 30 involved in aseptic meningitis cases in Brazil (1998–2008). J Med Virol 83: 2164–2171. 22012725 10.1002/jmv.22235

[16] Faustini A, Fano V, Muscillo M, Zaniratti S, La Rosa G, et al (2006) An outbreak of aseptic meningitis due to echovirus 30 associated with attending school and swimming in pools. Int J Infect Dis 10: 291–297. 16458563 10.1016/j.ijid.2005.06.008

[17] Starlin R, Reed N, Leeman B, Black J, Trulock E, et al (2001) Acute flaccid paralysis syndrome associated with echovirus 19, managed with pleconaril and intravenous immunoglobulin. Clin Infect Dis 33: 730–732. 11477532 10.1086/322624

[18] Alexander JP, Ehresmann K, Seward J, Wax G, Harriman K, et al (2009) Transmission of imported vaccine-derived poliovirus in an undervaccinated community in Minnesota. J Infect Dis 199: 391–397. 19090774 10.1086/596052

[19] Svitkin YV, Imataka H, Khaleghpour K, Kahvejian A, Liebig HD, et al (2001) Poly(A)-binding protein interaction with elF4G stimulates picornavirus IRES-dependent translation. Rna 7: 1743–1752. 11780631 PMC1370214

[20] Bergamini G, Preiss T, Hentze MW (2000) Picornavirus IRESes and the poly(A) tail jointly promote cap-independent translation in a mammalian cell-free system. Rna 6: 1781–1790. 11142378 10.1017/s1355838200001679 PMC1370048

[21] Brown B, Oberste MS, Maher K, Pallansch MA (2003) Complete genomic sequencing shows that polioviruses and members of human enterovirus species C are closely related in the noncapsid coding region. J Virol 77: 8973–8984. 12885914 10.1128/JVI.77.16.8973-8984.2003 PMC167246

[22] Zhang Y, Zhu S, Yan D, Liu G, Bai R, et al (2010) Natural type 3/type 2 intertypic vaccine-related poliovirus recombinants with the first crossover sites within the VP1 capsid coding region. PLoS ONE 5: e15300. 21203565 10.1371/journal.pone.0015300 PMC3006203

[23] Arita M, Shimizu H, Nagata N, Ami Y, Suzaki Y, et al (2005) Temperature-sensitive mutants of enterovirus 71 show attenuation in cynomolgus monkeys. J Gen Virol 86: 1391–1401. 15831951 10.1099/vir.0.80784-0

[24] Oberste MS, Maher K, Flemister MR, Marchetti G, Kilpatrick DR, et al (2000) Comparison of classic and molecular approaches for the identification of untypeable enteroviruses. J Clin Microbiol 38: 1170–1174. 10699015 10.1128/jcm.38.3.1170-1174.2000 PMC86366

[25] Oberste MS, Maher K, Kilpatrick DR, Pallansch MA (1999) Molecular evolution of the human enteroviruses: correlation of serotype with VP1 sequence and application to picornavirus classification. J Virol 73: 1941–1948. 9971773 10.1128/jvi.73.3.1941-1948.1999 PMC104435

[26] Nix WA, Oberste MS, Pallansch MA (2006) Sensitive, seminested PCR amplification of VP1 sequences for direct identification of all enterovirus serotypes from original clinical specimens. J Clin Microbiol 44: 2698–2704. 16891480 10.1128/JCM.00542-06 PMC1594621

[27] Oberste MS, Maher K, Michele SM, Belliot G, Uddin M, et al (2005) Enteroviruses 76, 89, 90 and 91 represent a novel group within the species Human enterovirus A. J Gen Virol 86: 445–451. 15659764 10.1099/vir.0.80475-0

[28] Smura TP, Junttila N, Blomqvist S, Norder H, Kaijalainen S, et al (2007) Enterovirus 94, a proposed new serotype in human enterovirus species D. J Gen Virol 88: 849–858. 17325357 10.1099/vir.0.82510-0

[29] Georgescu MM, Tardy-Panit M, Guillot S, Crainic R, Delpeyroux F (1995) Mapping of mutations contributing to the temperature sensitivity of the Sabin 1 vaccine strain of poliovirus. J Virol 69: 5278–5286. 7636970 10.1128/jvi.69.9.5278-5286.1995 PMC189363

[30] Zhang Y, Wang J, Guo W, Wang H, Zhu S, et al (2011) Emergence and transmission pathways of rapidly evolving evolutionary branch c4a strains of human enterovirus 71 in the central plain of china. PLoS One 6: e27895. 22125635 10.1371/journal.pone.0027895 PMC3220707

[31] Zhang Y, Yan D, Zhu S, Wen N, Li L, et al (2010) Type 2 vaccine-derived poliovirus from patients with acute flaccid paralysis in china: current immunization strategy effectively prevented its sustained transmission. J Infect Dis 202: 1780–1788. 21050127 10.1086/657410

[32] Lewthwaite P, Perera D, Ooi MH, Last A, Kumar R, et al (2010) Enterovirus 75 encephalitis in children, southern India. Emerg Infect Dis 16: 1780–1782. 21029544 10.3201/eid1611.100672 PMC3294525

[33] Avellon A, Rubio G, Palacios G, Casas I, Rabella N, et al (2006) Enterovirus 75 and aseptic meningitis, Spain, 2005. Emerg Infect Dis 12: 1609–1611. 17176588 10.3201/eid1210.060353 PMC3290945

[34] Tapparel C, Junier T, Gerlach D, Van-Belle S, Turin L, et al (2009) New respiratory enterovirus and recombinant rhinoviruses among circulating picornaviruses. Emerg Infect Dis 15: 719–726. 19402957 10.3201/eid1505.081286 PMC2687021

[35] Piralla A, Rovida F, Baldanti F, Gerna G (2010) Enterovirus genotype EV-104 in humans, Italy, 2008–2009. Emerg Infect Dis 16: 1018–1021. 20507762 10.3201/eid1606.091533 PMC3086237

[36] World Health Organization. (2004) Polio laboratory manual, 4th edition. Document World Health Oraganization/IVB/04.10. Geneva, Switzerland.

[37] Zhang Y, Tan XJ, Wang HY, Yan DM, Zhu SL, et al (2009) An outbreak of hand, foot, and mouth disease associated with subgenotype C4 of human enterovirus 71 in Shandong, China. J Clin Virol 44: 262–267. 19269888 10.1016/j.jcv.2009.02.002

[38] Sun Q, Zhang Y, Zhu S, Cui H, Tian H, et al (2012) Complete genome sequence of two coxsackievirus A1 strains that were cytotoxic to human rhabdomyosarcoma cells. J Virol 86: 10228–10229. 22923792 10.1128/JVI.01567-12 PMC3446563

[39] Tamura K, Peterson D, Peterson N, Stecher G, Nei M, et al (2011) MEGA5: molecular evolutionary genetics analysis using maximum likelihood, evolutionary distance, and maximum parsimony methods. Mol Biol Evol 28: 2731–2739. 21546353 10.1093/molbev/msr121 PMC3203626

[40] Kumar S, Nei M, Dudley J, Tamura K (2008) MEGA: a biologist-centric software for evolutionary analysis of DNA and protein sequences. Brief Bioinform 9: 299–306. 18417537 10.1093/bib/bbn017 PMC2562624

[41] Lole KS, Bollinger RC, Paranjape RS, Gadkari D, Kulkarni SS, et al (1999) Full-length human immunodeficiency virus type 1 genomes from subtype C-infected seroconverters in India, with evidence of intersubtype recombination. J Virol 73: 152–160. 9847317 10.1128/jvi.73.1.152-160.1999 PMC103818

[42] Blomqvist S, Bruu AL, Stenvik M, Hovi T (2003) Characterization of a recombinant type 3/type 2 poliovirus isolated from a healthy vaccinee and containing a chimeric capsid protein VP1. J Gen Virol 84: 573–580. 12604808 10.1099/vir.0.18708-0

[43] Yang CF, Naguib T, Yang SJ, Nasr E, Jorba J, et al (2003) Circulation of endemic type 2 vaccine-derived poliovirus in Egypt from 1983 to 1993. J Virol 77: 8366–8377. 12857906 10.1128/JVI.77.15.8366-8377.2003 PMC165252

